# Prospect of research on anti-atherosclerosis effect of main components of traditional Chinese medicine Yiqi Huoxue Huatan recipe through gut microbiota: A review

**DOI:** 10.1097/MD.0000000000037104

**Published:** 2023-02-02

**Authors:** Hongtao Huang, Hanjun Zhao, Lv Wenqing, Feiyue Xu, Xiaolong Wang, Yili Yao, Yu Huang

**Affiliations:** a Department of Cardiology, Shanghai Gongli Hospital, The Second Military Medical University, Shanghai, China; b Shuguang Hospital Affiliated to Shanghai University of Traditional Chinese Medicine, Shanghai, China; c Shanghai Pudong New District Pudong Hospital, Shanghai, China.

**Keywords:** atherosclerosis, gut microbiota, mechanism of action, traditional Chinese medicine YHHR

## Abstract

The incidence and mortality rates of cardiovascular diseases are on the rise globally, posing a severe threat to human health. Atherosclerosis (AS) is considered a multi-factorial inflammatory disease and the main pathological basis of cardiovascular and cerebrovascular diseases, as well as the leading cause of death. Dysbiosis of the gut microbiota can induce and exacerbate inflammatory reactions, accelerate metabolic disorders and immune function decline, and affect the progression and prognosis of AS-related diseases. The Chinese herbal medicine clinicians frequently utilize Yiqi Huoxue Huatan recipe, an effective therapeutic approach for the management of AS. This article reviews the correlation between the main components of Yiqi Huoxue Huatan recipe and the gut microbiota and AS to provide new directions and a theoretical basis for the prevention and treatment of AS.

## 1. Introduction

Atherosclerosis (AS) is a chronic progressive vascular disease that involves several mechanisms. Firstly, endothelial cell injury is one of the critical factors in the development of atherosclerosis.^[1]^ Factors such as inflammation, oxidative stress, and high cholesterol levels can all cause damage to endothelial cells, disrupting their normal barrier function.^[2]^ Subsequently, elevated cholesterol levels lead to the accumulation of low-density lipoprotein (LDL) within the vascular wall, which is oxidized into oxidized LDL and then engulfed by macrophages to form foam cells. The accumulation of foam cells ultimately evolves into mature atherosclerotic plaques.^[3]^ During this process, the exposed collagen and other coagulation factors on the surface of ruptured plaques can activate platelets, promoting platelet adhesion and aggregation to form thrombi, leading to cardiovascular events.^[4]^ Multiple complex biological processes are involved in the mechanisms of atherosclerosis, including endothelial cell injury, lipid deposition, platelet activation, and coagulation, as well as inflammatory reactions.

AS provides an essential pathologic basis for cardiovascular diseases, which can lead to a variety of vascular diseases, including myocardial infarction, stroke, and disabling peripheral artery disease, resulting in more complications and mortality.^[2]^ The precise pathogenesis of AS remains unclear, thereby impeding the clinical management of this condition.^[5]^ Previous studies on AS have mainly focused on inflammation, lipid metabolism, and other aspects. In contrast, recent studies suggested that arterial plaques in AS patients also contain bacterial DNA, and the same bacterial populations in AS plaques have also been discovered in the gut.^[6,7]^ As a chronic inflammatory disease, AS is closely related to gut microbiota (GM).^[8,9]^ Dysbiosis of the GM can trigger and aggravate inflammatory reactions, leading to AS.^[10,11]^ The GM plays a vital role in regulating the immune system. An imbalance in GM can result in immune dysfunction, further promoting the development of AS.^[12,13]^ Research indicates that the gut microbiome (GM) of individuals with AS differs from that of healthy individuals. Individuals with AS exhibit decreased GM diversity and abundance, as well as an elevated proportion of pathogenic bacteria.^[14,15]^ Dental plaque and bacteria colonizing the gut *Firmicutes Veillonella, Streptococcus*, and *Chlamydia* had been found in AS plaque.^[16,17]^ It has also been reported that *Porphyromonas gingivalis* accelerates AS lesion development through toll-like receptors (TLR)-2-mediated mechanisms^[18]^ and increases foam cell formation in infected macrophages.^[19]^ In addition, studies have found that AS patients have elevated levels of lipopolysaccharides (LPS) in the gut, and LPS can activate the immune system and promote inflammation.^[20,21]^ Some studies have indicated that GM can directly or indirectly influence the development of AS through genetic and environmental factors.^[22]^ The above results demonstrate that the bacteria in AS plaques originate from GM and can affect plague stability and promote cardiovascular disease progression.

Traditional Chinese medicine has been proven to regulate GM and improve symptoms related to atherosclerosis. Studies have demonstrated that various anti-AS traditional Chinese herbal monomers, extracts, and compounds can prevent and treat AS by regulating GM. For example, berberine, the active ingredient of *Coptis chinensis*, exhibits poor water solubility and low bioavailability, making it difficult to be absorbed through the intestinal tract. However, studies have shown that GM can metabolize berberine into demethoxy products and reduce products, improving its absorption and utilization by the body.^[23]^ Wang et al discovered through studies that *Polygonatum odoratum* polysaccharide enhanced GM species abundance and improved GM structure in rats fed a high-fat diet (HFD) and increased the level of short-chain fatty acids (SCFA), thereby up-regulating the expression of the genes involved in adipocyte differentiation and down-regulating the expression of proteins related to lipid synthesis, ultimately inhibiting lipid metabolism and AS.^[24]^

Yiqi Huoxue Huatan recipe (YHHR) is a complex herbal formulation in traditional Chinese medicine. YHHR is composed of several main components, including *C chinensis, Astragalus membranaceus, Salvia miltiorrhiza*, and leeches. Many previous studies have demonstrated the anti-inflammatory and anti-AS effects and mechanisms of YHHR.^[25–28]^ However, no study has yet demonstrated the effect of YHHR on AS through GM. In this paper, the correlation of YHHR and its main components with GM and AS was summarized to provide a reference for further study of traditional Chinese medicine and the development of innovative traditional Chinese medicine targeting GM.

## 2. GM and atherosclerosis

The progression of AS is positively correlated to the inflammatory response. Recent studies have shown that changes in the composition and diversity of the GM are closely associated with chronic low-grade inflammation in tissues and the occurrence of AS.^[29]^ The increase in intestinal pathogens exacerbates the inflammatory response, increases the body secretion of inflammatory factors, and damages the vascular endothelium, thereby increasing the risk of heart disease.^[30]^ Karlsson et al^[31]^ performed metagenomic sequencing of GM in patients with cerebrovascular events caused by carotid stenosis and discovered that the number of GM *Colinsella* increased significantly in these patients while the numbers of *Roseburia* and *Eubacterium* decreased, indicating that AS is closely related to gut dysbiosis. Koren et al utilized sequencing of genomic material to demonstrate the bacterial composition present in atherosclerotic plaques, as well as the oral microbiota. Their findings revealed that Chrysomonas was present and dominated by *Veillonella* and *Streptococcus*. Furthermore, 2 unknown bacteria genera belonging to *Erysipelotrichaceae* and *Lachnospiraceae* were positively correlated with total cholesterol and LDL, indicating that GM may affect cholesterol metabolism and be involved in the occurrence of AS.^[32]^

## 3. Metabolites of GM and atherosclerosis

In addition to direct interaction with the host metabolism and immune system, GM can also influence the occurrence and development of AS through its metabolites. Increasing evidence suggests that GM and its metabolites, such as SCFA and trimethylamine oxide (TMAO), play a role in AS by regulating inflammation and metabolism of lipids, cholesterol, and glucose.^[33]^ Studies have demonstrated that SCFAs are metabolites produced by GM through fermentation and decomposition of starch and dietary fiber in food, mainly including acetic acid, propionic acid, and butyric acid, which are high in content in the intestinal lumen and can maintain intestinal homeostasis, playing a role in anti-AS.^[6]^ Wherein, by regulating the receptor signaling pathways, propionate and butyrate can affect the immune and metabolic functions of the body and inhibit NF-κB and tumor necrosis factor (TNF) signaling pathways, leading to a decline in the expression of vascular cell adhesion molecule-1 and intercellular adhesion molecule-1 synthesized by oxidized LDL-activated vascular endothelial cells, thereby inhibiting the occurrence of AS.^[34,35]^ Some studies have also demonstrated that SCFAs can not only maintain the acidic environment of the gut and inhibit the growth of harmful bacteria in the gut but also bind to the GPR43 receptor (G protein-coupled receptor 43) to stimulate L cells to regulate energy utilization, improve metabolism, and regulate immunity.^[36]^

In recent years, the relationship between TMAO and AS plaques has received much attention.^[37,38]^ The generation of TMAO is inseparable from the involvement of GM. Bogiatzi et al^[39]^ show that intestinal bacterial metabolites such as TMAO, tolylglucosidic acid, and phenylacetylglutamine (PAGln) are all connected to AS. The influence of TMAO on the formation of AS may be through the following aspects: Specific microbial enzymes can produce trimethylamine (TMA) through nutrients such as choline, l-carnitine and lecithin, and then hinder the reverse transport of cholesterol after oxidized into TMAO in the liver;^[40]^ TMAO can promote the expression of Class B scavenger receptor, that is, CD36 and SRA on the cell surface, thereby increasing the formation of foam cells;^[41]^ TMAO inhibits the expression of bile acid synthase CYP7A1 and CYP27A1 and bile acid transporter in the liver^[42]^ and suppresses bile synthesis to affect the cholesterol clearance from the body. However, there is also literature stating that TMAO negatively correlates with the formation of AS. TMAO can inhibit the formation of AS by inhibiting the reabsorption of cholesterol and reducing the blood cholesterol concentration.^[43,44]^

## 4. Mechanism of GM to regulate atherosclerosis

GM can affect the occurrence and development of AS in various ways. First, bacterial infections activate the immune system, causing harmful inflammatory responses. Local infections and distant infections caused by bacterial invasion of AS plaques can promote the development of AS. In addition, infection leads to an increase in pro-inflammatory cytokines and chemokines, which may be mediated by TLR 4 expressed in macrophages.^[45–47]^ LPS derived from the dysbiosis of GM can regulate TLRs and their downstream MyD88 and NF-κB and promote the generation of cytokines such as IL-6, IL-1, IL-27, and TNF-α, thereby increasing the risk of AS.^[48]^ Second, the microbiota can affect AS by regulating cholesterol metabolism, as they can change the serum cholesterol level.^[49]^ GM is also essential in modifying bile acids, which are signal molecules initiating downstream signaling pathways by binding to farnesoid X receptor (FXR). Activation of FXR induces flavin monooxygenase 3 activity in the liver, which converts TMA into TMAO.^[50–53]^ Some studies also suggest that specific dietary components and microbial metabolites can produce beneficial and harmful molecules^[54,55]^ that may promote the development of AS. In recent years, there have been studies suggesting that the GM and its metabolites can regulate adrenaline levels, potentially leading to cardiovascular diseases. Chen discovered that a GM-dependent metabolite called PAGln can increase the risk of atherogenesis by transmitting signals through adrenergic receptors.^[56]^ Nemet et al^[57]^ found a positive correlation between plasma levels of PAGln and platelet aggregation function, as well as incidental thrombotic events. This metabolite is produced when the GM metabolizes polyacrylic acid, which is easily absorbed by the hepatic portal vein system and then metabolized in the human liver.

In terms of treatment, there is evidence to suggest that the GM plays a regulatory role in preventing and treating atherogenesis. Several studies have indicated that bifidobacteria in the gut have an inhibitory effect on atherogenesis. Liao et al^[58]^ found that tea polyphenols can influence gut bifidobacteria, thereby affecting the mechanism of atherogenesis and improving blood lipid levels in the observation group. *Bifidobacterium* displayed an effect of decreasing visceral fat mass in people,^[59]^ subsequently ameliorating the progression of AS and mitigating effects on HFD, an increased Muribaculaceae level accompanied by induced obesity.^[60]^ Bifidobacteria and the gut microbiome produce SCFA to lower gut pH, form a biological barrier, and secret antibacterial compounds to weaken harmful bacteria.^[61]^ SCFAs have been found to inhibit LPL activity by activating PPARγ and upregulating ANGPTL4 levels, which leads to the regulation of fatty acid oxidation in muscle and fat cells, thereby reducing fat storage.^[62–64]^ Therefore, regulating dietary patterns of *Bifidobacterium* intestinalis (IB) may further influence the development of AS.

Furthermore, targeted recombination of the GM can treat atherogenesis by altering the chemical environment in the intestines. Chen et al^[65]^ discovered that orally administering cyclic d,l-α-peptides, selective modifiers of bacterial growth, can improve intestinal barrier integrity and suppress the production of various pro-inflammatory cytokines and chemokines. Recombined GM can also downregulate the fibroblast growth factor 15 endocrine axis of the FXR, increase the expression of Cyp7A1, and consequently lower plasma cholesterol levels, thereby decreasing the formation of atherogenesis.

### 4.1. Atherosclerosis treatment through regulation of GM by traditional Chinese medicine

Traditional Chinese medicine and its compounds are predominantly by oral drugs and come in direct contact with GM to take effect. Two metabolites can be produced in this process, including GM metabolites and GM-transformed traditional Chinese herbal compounds. Previous studies have made it clear that GM plays an essential role in maintaining balance within the body.^[66,67]^ Traditional Chinese medicine can improve cardiovascular diseases through the regulation of GM,^[68]^ the effects of which are mainly manifested in 3 aspects: Regulation of the composition of GM; regulation of the metabolism of GM; GM can metabolize the active ingredients in traditional Chinese medicine. The main components of YHHR, that is, *C chinensis, A membranaceus, S miltiorrhiza*, and leeches are presented below as examples.

### 4.2. Possible mechanism of *C chinensis* regulating atherosclerosis through GM

**Figure 1.**
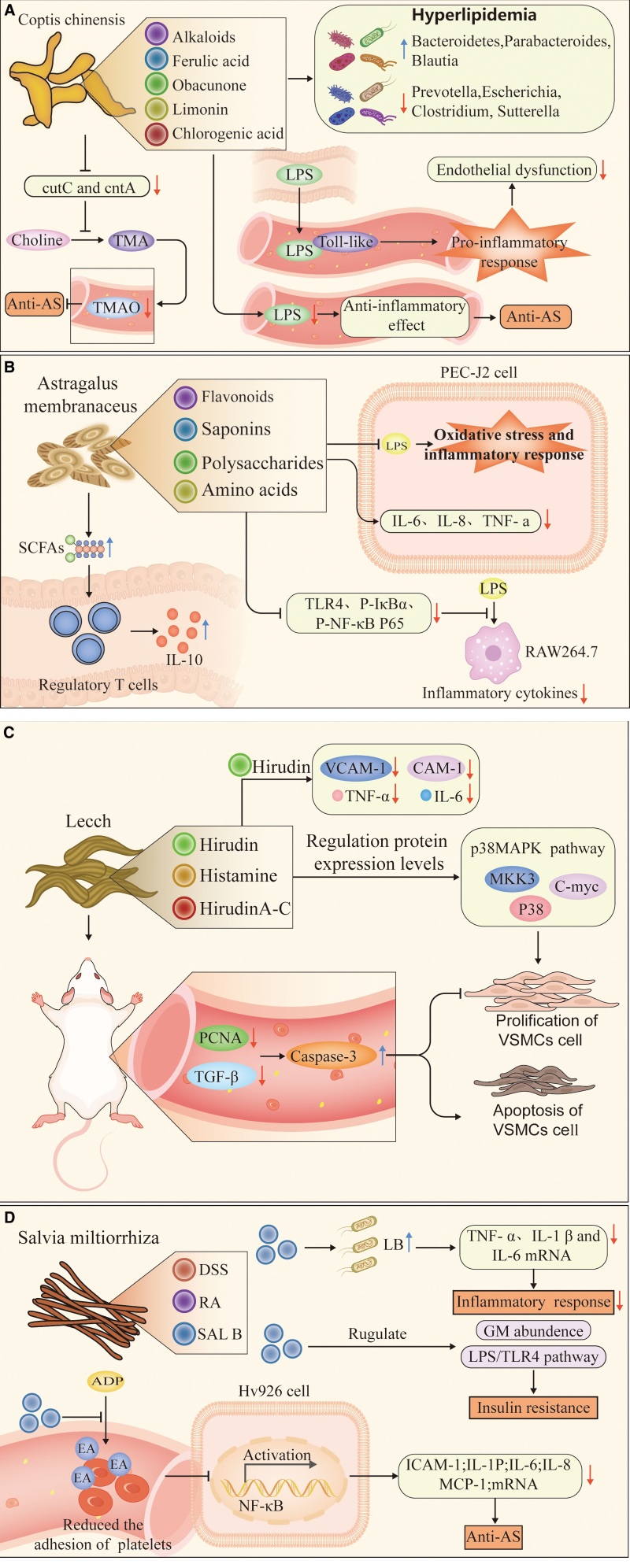
(A) Mechanism of *Coptis chinensis* regulating atherosclerosis through gut microbiota. (B) Mechanism of *Astragalus membranaceus* regulating atherosclerosis through gut microbiota. (C) Mechanism of leech regulation of atherosclerosis via gut microbiota. (D) Mechanism of *Salvia miltiorrhiza* regulating atherosclerosis through gut microbiota.

*C chinensis* is mainly composed of alkaloids, ferulic acid, obacunone, limonin, chlorogenic acid, and other components, the alkaloids of which mainly include berberine, coptisine, palmatine, jatrorrhizine, and magnoflorine, with berberine being the main anti-bacterial active ingredient with higher content.^[69]^ Studies proved that *C chinensis* increase the abundance of *Bacteroidetes, Parabacteroides* and *Blautia*, also eliminate the number of *Prevotella, Escherichia, Clostridium*, and *Sutterella* in hyperlipidemia patients.^[70]^ Berberine hydrochloride treatment decreases the number of *Ruminococcus gnavus* (Mediterranean bacillus), *Ruminococcus shinkii* (blautia), *Lactobacillus acidophilus* (lactobacillus), *Lactobacillus lactis* (lactobacillus), and *Lactococcus lactis* (lactococcus) in the colon and ileum of C57BL/6 mice^[71]^ (Fig. 1A). Studies have indicated that the regulation of GM, especially the abundance of *Akkermansia*, contributes to the anti-AS and metabolic protective effects of berberine by inhibiting intestinal inflammation and promoting the integrity of the intestinal epithelial barrier.^[72]^ Furthermore, coptisine can also manipulate GM by inhibiting the overgrowth of *Enterobacter cloacae*, lowering the content of LPS in inflammatory states, and reducing the systemic inflammatory response, thereby delaying the development of AS.^[73]^ Berberine has been proven to affect the SCFAs-producing bacteria, which can enrich the population of butyrate-producing bacteria in the GM, thereby promoting the synthesis of butyrate through the acetyl-coenzyme a-butyrate coenzyme a-butyrate pathway and reducing the blood lipid and glucose levels.^[74,75]^ SCFAs-producing bacteria can benefit the host by protecting the mucosa from pathogen-induced damage, providing nutrients to colon cells, and alleviating inflammation.^[71]^

Studies have found that berberine can reduce TMA/TMAO production in C57BL/6J and ApoE KO mice and reduce AS lesion area in ApoE KO mice. The primary mechanism is to reduce the conversion of choline to TMA by decreasing functional gene levels of the critical genes cutC and cntA in the TMA synthesis pathway and ultimately reduce serum TMAO level, thereby inhibiting the development of AS^[76]^ (Fig. 1A).

Serum LPS is a metabolite of GM transformation. When LPS crosses the gut barrier and enters the bloodstream, it binds to TLRs to initiate the host pro-inflammatory response, leading to endothelial dysfunction and the progression and vulnerability of AS plaques (Fig. 1A). Some studies suggested that berberine can lower the level of plasma LPS to produce an anti-inflammatory effect, thereby inhibiting arterial plaque formation.^[72,77]^

### 4.3. Possible mechanism of *A membranaceus* regulating atherosclerosis through GM

*A membranaceus* is composed of flavonoids, saponins, polysaccharides, amino acids, and other compounds, the flavonoids of which mainly include quercetin, isorhamnetin, calycosin, and formononetin, while the saponins mainly contain hederasaponin.^[78]^ Modern pharmacological studies have exhibited that *A membranaceus* can enhance immune function, dilate blood vessels, improve myocardial contractility, and inhibit thrombosis through a variety of pathways and targets, which are widely used in the treatment of AS diseases.^[75]^

At the phylum level, *A membranaceus* increases the relative abundance of *Bacteroidetes*, reduces the relative abundance of Firmicutes, and increases the ratio of Bacteroidetes to Firmicutes. The increased Bacteroidetes can decompose plant sugars into prebiotics and reduce inflammation. In contrast, the increase in the ratio of Bacteroidetes to Firmicutes directly affects the metabolism of dietary fiber by GM. It increases the concentration of SCFAs, where SCFAs can induce the production and differentiation of regulatory T cells in the intestine, thereby promoting the production of the anti-inflammatory factor IL-10 and playing an anti-inflammatory role (Fig. 1B). At the genus level, *A membranaceus* increases the number of beneficial bacteria *Bifidobacteria*, improves endotoxemia in mice, protects the intestinal mucosal barrier, reduces LPS load and inflammatory signaling pathways triggered by increased LPS, and lowers the concentrations of pro-inflammatory factors IL-1 in serum β, IL-6, and TNF-α.^[79,80]^

It is shown through study that Astragalus polysaccharides significantly inhibited the oxidative stress and inflammatory response of LPS-induced intestinal epithelial cells IPEC-J2 and significantly down-regulated the expression of IL-6, IL-8 and TNF-α while significantly up-regulated the expression of IL-10 gene^[81]^ (Fig. 1B). In the inflammation model of LPS-induced macrophage RAW264.7, *A membranaceus* significantly down-regulated the expression of TLR4, P-IκBα, and P-NF-κB P65 proteins, leading to a significant decrease in the level of inflammatory cytokines^[82]^ (Fig. 1B). All the above studies have demonstrated that *A membranaceus* can inhibit the development of AS by regulating GM and its metabolites.

### 4.4. Possible mechanism of leech regulation of atherosclerosis via GM

Leech is one of the earliest records of traditional Chinese medicines in *Shennong Herbal Classics*, the main ingredients of which are hirudin, histamine, and hirudin A-C. It has been extensively validated as a symbolic biological feature of the thrombin inhibitor.^[83]^ Meanwhile, the leech has effects such as anti-coagulant, anti-thrombotic, anti-inflammatory, anti-fibrosis, and anti-tumor^,[84,85]^ as well as the preventive effect on ischemic diseases.^[86,87]^ Some studies indicated that the leech in traditional Chinese medicine can regulate and lower the TGF-β 1 and PCNA in the serum of rats to inhibit the proliferation of VSMCs while upregulating the expression of Caspase-3 to promote the apoptosis of VSMCs. It can also regulate the expression levels of p38MAPK signaling pathway proteins MKK3, P38, and C-myc to regulate the proliferation and apoptosis of VSMCs and affect the AS progression^[88]^ (Fig. 1C). Liu et al discovered that hirudin can inhibit angiotensin II-induced hypertrophy and death of H9c2 cells and significantly reduce mRNA and protein expression levels of STAT3, MAPK1, and IL-6^[89]^ (Fig. 1C). Studies show that hirudin and hirudin A-C act on the E1 component of pyruvate dehydrogenase subunit α [EC: 1.2.4.1], hydroxy glutathione hydrolase [EC: 3.1.2.6], and Acetyl-CoA acetyltransferase [EC: 2.3.1.9], thereby influencing pyruvic acid metabolism and reducing taurine content, playing a role in anti-oxidation.^[90]^ Hirudin can reduce the expression of vascular cell adhesion molecule-1 and intercellular adhesion molecule-1 protein in rat myocardium and TNF-α and IL-6 levels in serum and weaken cell adhesion by inhibiting the inflammatory reaction,^[91]^ which may play a role in anti-AS (Fig. 1C). Although leeches and their main components show apparent effects in terms of AS, anti-coagulant, and anti-inflammatory, to date, there has not been any study on the relationship between leeches and GM.

### 4.5. Possible mechanism of *S miltiorrhiza* regulating atherosclerosis through GM

The active ingredients in *S miltiorrhiza* are mainly hydrophilic aromatic acids and lipophilic tanshinones, with the former represented by ingredients including salviamiltiorrhizasu, rosmarinic acid and salvianolic acid B (SAL B), and the latter represented by compounds including tanshinone I, tanshinone IIA, cryptotanshinone and dihydrotanshinone I.^[92]^

Previous studies demonstrated that SAL B reduces the levels of serum cTnI, CK-MB, and MDA and increases the levels of serum NO and SOD in the ischemia-reperfusion rat model, so it affects anti-ischemia-reperfusion myocardial injury.^[93]^ During in vitro experiments, SAL B reduced the adhesion of ADP-activated platelets to EA.Hy926 cells inhibited the activation of NF-kB and reduced the protein and mRNA levels of pro-inflammatory factors IC AM-1, IL-1P, IL-6, IL-8, and MCP-1, showing an anti-AS effect^[94]^ (Fig. 1D).

The main active ingredients of *S miltiorrhiza* play an anti-inflammatory role by regulating the species and abundance of GM. It is demonstrated through study that polysaccharides in SAL B can regulate the production of probiotic LB and lower the mRNA concentrations of proinflammatory cytokines (TNF-α, IL-1 β, and IL-6) to slow down the process of inflammation (Fig. 1D). At the same time, SAL B can reduce the abundance of cyanobacteria at the phylum level and increase the *Bacteroidetes*/*Firmicutes* ratio at the genus level, which is one of the biomarkers of obesity and type 1 diabetes.^[95]^ Studies have also found that Sal B can down-regulate TLR4 and myoid differential factor-88, attenuating weight gain and insulin resistance by regulating GM abundance and the LPS/TLR4 signaling pathway in obese mice^[96]^ (Fig. 1D).

In addition, *S miltiorrhiza* and its main active ingredients also confirmed to reduce the body inflammatory response and the metabolic process by regulating the metabolism of GM. Zhuo et al^[97]^ discovered through study that the total phenolic acids in the stems and leaves of *S miltiorrhiza* and the total phenolic acids in the roots of *S miltiorrhiza* can regulate gut dysbiosis in mice of the model, and they both can increase the content of SCFAs in the intestine by regulating the number of SCFA-producing bacteria. Salvianic borneol ester is a synthetic derivative of the natural compound used in Compound Danshen Dropping Pills, which has the effect of inhibiting LPS-induced inflammation and macrophage lipid accumulation and can reduce obesity, insulin resistance, hepatic steatosis, and low-grade systemic inflammation in the mice fed with HFD by regulating the GM.^[98]^ Some studies have found that *Astragalus-S miltiorrhiza* may have a role in anti-AS by changing the structure and composition of GM, improving the abundance and diversity of GM, increasing the abundance of probiotics such as *Lactobacillus* and *Bifidobacterium*, enhancing the intestinal mucosal barrier and immune function, inhibiting the body inflammatory response, and reducing the blood lipids.^[99]^

## 5. Conclusion and prospect

Numerous studies have indicated a close correlation between dysbiosis of GM and AS. On the one hand, GM directly regulates the metabolism of blood lipids and blood glucose, disrupts the body metabolic balance to promote the process of AS, and affects the process of AS through inflammatory response. On the other hand, it can further influence the occurrence and development of AS by regulating its metabolites, such as SCFA and TMAO. Furthermore, the GM plays a crucial role in regulating the immune system, and dysbiosis of the GM can lead to immune dysfunction, further promoting the development of AS. AS treatment now targets the regulation of gut dysbiosis. Previous studies have demonstrated that YHHR has anti-inflammatory and anti-AS effects, which may be related to its influence on GM metabolism of traditional Chinese medicine and its related active ingredients. This improvement of the absorption and utilization contribute to increase probiotics, and reduce harmful bacteria. Traditional Chinese medicine can regulate GM imbalance and prevent atherosclerosis potentially. Some studies have shown that YHHR can regulate inflammation by improving the composition and function of GM, reducing inflammation and vascular damage in atherosclerosis. For example, herbs like *S miltiorrhiza*, Astragalus and Coptis have been studied and found to have antioxidant, anti-inflammatory, and lipid-lowering effects, which can reduce the risk of atherosclerosis by modulating GM.

This may be attributed to the involvement of GM in the metabolism of traditional Chinese medicine and its related bioactive compounds, improving the absorption and utilization of YHHR in the body, increasing probiotics, and reducing harmful bacteria.

However, despite the potential of traditional Chinese medicine in this area, related research is still in its early stages. Further in-depth studies are needed to validate the regulatory effects of YHHR on GM imbalance and atherosclerosis. With the further development of the pertinent research, we will gain more insight into the interaction between GM, AS, and traditional Chinese medicine YHHR and provide a scientific basis for the anti-AS effect of traditional Chinese medicine YHHR.

## Author contributions

**Data curation:** Feiyue Xu.

**Funding acquisition:** Xiaolong Wang, Yili Yao.

**Investigation:** Xiaolong Wang.

**Methodology:** Feiyue Xu.

**Project administration:** Yu Huang, Lv Wenqing, Yili Yao.

**Resources:** Feiyue Xu, Xiaolong Wang, Yili Yao.

**Software:** Lv Wenqing.

**Supervision:** Yu Huang.

**Visualization:** Lv Wenqing.

**Writing – original draft:** Yu Huang, Hongtao Huang, Hanjun Zhao.

**Writing – review & editing:** Yu Huang, Hongtao Huang, Hanjun Zhao.
